# Colonization of the bovine uterus by *Candida kefyr*

**DOI:** 10.1186/s13028-017-0329-5

**Published:** 2017-09-16

**Authors:** Cecilia Christensen Karstrup, Bent Aalbæk, Kirstine Klitgaard, Tim Kåre Jensen, Hanne Gervi Pedersen, Jørgen Steen Agerholm

**Affiliations:** 1 Department of Veterinary Clinical Sciences, Faculty of Health and Medical Sciences, University of Copenhagen, Dyrlægevej 68, 1870 Frederiksberg, Denmark; 2 Department of Veterinary and Animal Sciences, Faculty of Health and Medical Sciences, University of Copenhagen, Grønnegårdsvej 15, 1870 Frederiksberg, Denmark; 3 National Veterinary Institute, Technical University of Denmark, Kemitorvet, Building 202, 1870 Kgs. Lyngby, Denmark

**Keywords:** Aetiology, Endometritis, Fungus, *Kluyveromyces marxianus*, Mycosis, Postpartum

## Abstract

**Background:**

While fungal infections of the bovine uterus are well-known diseases in pregnant cattle, very limited knowledge exists on the presence and significance of fungi in the uterus of non-pregnant cows. Presence of fungi in the uterine lumen of postpartum (pp) cows has been reported, but little attention has been paid to this as most studies of the bovine pp uterus have focused on bacteria.

**Case presentation:**

Microscopy of uterine lavage cytology slides of three cows from one herd revealed the presence of numerous yeast-like organisms, which were located either free in the fluid or within macrophages. Two of the cows were around 30 days pp, while the third was 7 months pp. None of the cows had been treated with antibiotics. Culturing of the flush samples was unsuccessful, but Sanger sequencing of DNA extracted from an endometrial biopsy of one of the cows revealed the presence of *Candida kefyr* (*Kluyveromyces marxianus*). Fluorescence in situ hybridization examination of endometrial tissue sections of two cows using probes targeting 18S rRNA of the *K. marxianus* group was performed and revealed the presence of yeast cells on the endometrium. Histology was performed and demonstrated hyphal and non-hyphal yeast-like organisms on the surface of endometrium and in the crypts. Tissue invasion was restricted to the superficial part of the epithelium and although endometrial inflammation was present, this was mild and considered as not being caused by the fungi. One of the cows became pregnant and delivered a normal calf at term, while the two others were not bred.

**Conclusions:**

*Candida kefyr* is commonly isolated from milk of cows with mastitis, but has not been reported in association with other diseases of cattle. The infection was present as a monoculture in all three cows, but the fungi had only colonized the uterine lumen and the endometrial surface. Only a mild non-suppurative endometrial inflammation was present, but within the uterine luminal content, many macrophages having phagocytized yeast cells were present. Re-examination of the cows did not reveal a persistent infection, so the infection probably resolved spontaneously.

**Electronic supplementary material:**

The online version of this article (doi:10.1186/s13028-017-0329-5) contains supplementary material, which is available to authorized users.

## Background

Fungal infections of the bovine uterus are well-known conditions in pregnant animals, where they are associated with sporadic abortions. Such infections first become established in the placentomes after haematogenous spread from a primary extrauterine focus but progression of the infection may lead to colonization of the interplacentomal chorioallantoic membrane and in some cases also of the amniotic fluid thus leading to foetal infection. The endometrium may also become infected but endometrial lesions are less severe than those of the foetal membranes [1]. In contrast to the well-described pathology of bovine mycotic abortion, very limited knowledge exists on the presence and significance of fungi in non-pregnant cows.

In the early postpartum (pp) period, the uterine lumen of most cows becomes contaminated by a mixed microflora ascending through the cervical canal; the outcome of which depends on the balance between the immunological capacity of the cow and the infection pressure. This uterine pp flora has been examined in several studies by culturing [2–4] and more recently by culture-independent methods [5–7] revealing a mixed flora, including a number of opportunistic pathogenic bacteria such as *Fusobacterium necrophorum* and *Trueperella pyogenes*. Studies of the pp uterine microflora of cattle have mainly focused on bacteria, but presence of fungi has also been mentioned, although not dealt with in detail [2].

Here we report the presence of *Candida kefyr* (*Kluyveromyces marxianus* [8]) in the uterus of three cows.

## Case presentations

Two cows (Nos. 1 and 2) were identified during another study on pp uterine infections performed in a Danish dairy herd with 1230 Holstein cows. Herd data, methodology and overall findings have been reported previously [9]. The third case was an infertile cow in the same herd that was examined due to a request from the farmer. The cows were of parity 4, 2 and 1, respectively, and had calved with viable calves and had had an uncomplicated pp course. Cow Nos. 1 and 2 had been examined 6 and 8 days pp, respectively, as part of the overall study and an endometrial biopsy and a uterine lavage sample had been taken and examined as previously reported [9]. Cow No. 1 was diagnosed with a uterine *T. pyogenes* infection at 6 days pp, but fungi were not observed in the uterus of neither of the two cows at that stage. The results of the laboratory examinations are reported in Additional file 1.

Cow Nos. 1 and 2 were re-examined at pp day 27 and 29, respectively, according to the overall study design while cow No. 3 was presented for the first time 223 days pp. Cow Nos. 1 and 2 had not yet been inseminated, while cow No. 3 had remained non-pregnant despite seven inseminations. Cow No. 1 had been treated for parturient paresis on pp day 22 while cow No. 3 had been treated for ketosis on pp day 6. None of the cows had received any treatment with antibiotics between calving and date of the examination.

All cows underwent a thorough gynaecological examination, including an inspection of the vulva, vaginal exploration and transrectal palpation of the reproductive organs. The cows were clinically unremarkable. The three cows were subjected to a uterine lavage from which the recovered fluid was used to prepare cytology slides and an endometrial biopsy was taken from the dorsal aspect of the uterine body as previously described in detail [9]. The endometrial biopsy was divided into two parts of which one was fixed in 10% neutral buffered formalin for histology and one placed in 1 mL RNAlater (Ambion, Austin, TX, USA) for DNA extraction and Sanger sequencing.

**Fig. 1:**
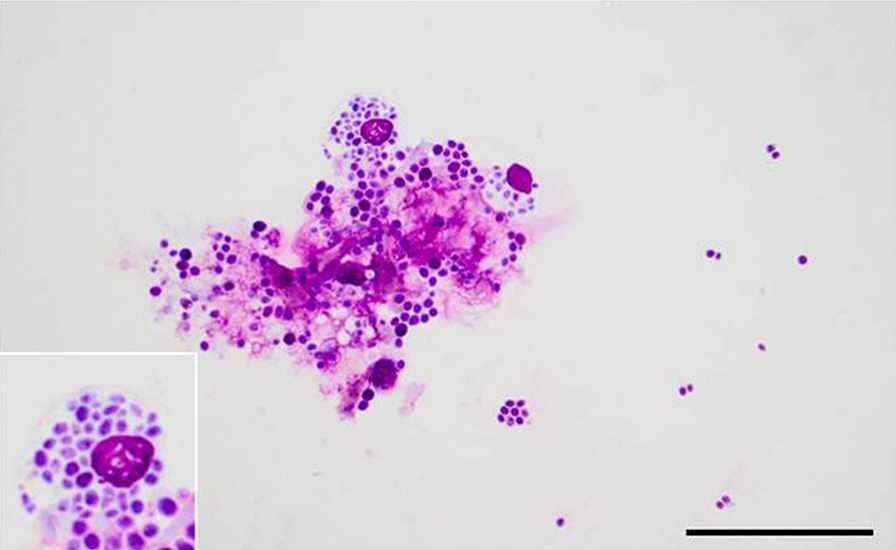
Photomicrograph of a cytology smear of a uterine lavage sample. Multiple *Candida kefyr* organisms are present both as isolated cells and as clusters of two or more cells. A conglomerate of organisms, mucus and macrophages, which have phagocytosed multiple organisms (insert), is present as well. Cow No. 3, Hemacolor^®^, bar = 50 µm

**Fig. 2:**
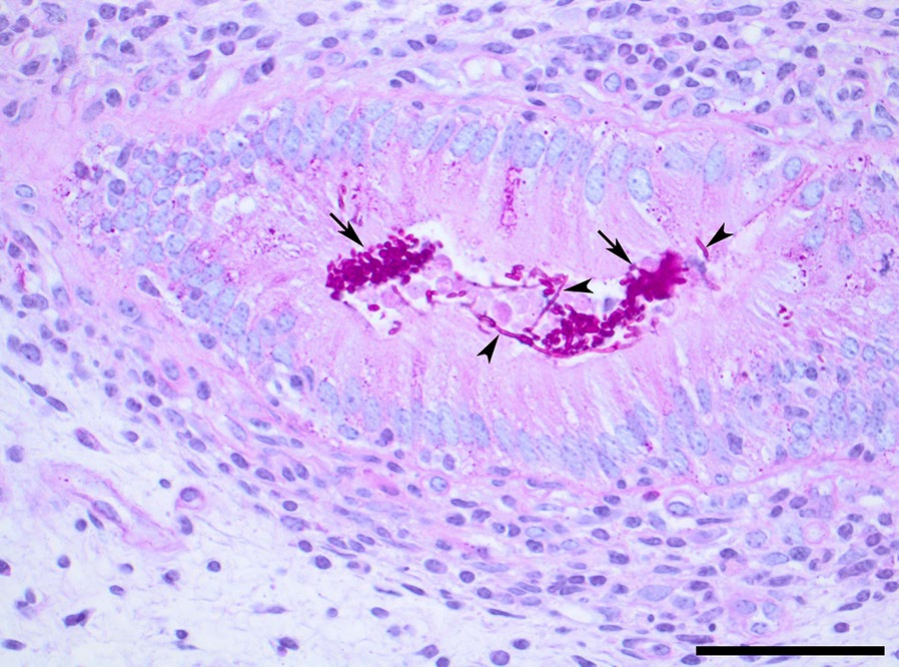
Photomicrograph of an endometrial biopsy. Clusters of *Candida kefyr* organisms are located in the lumen of an endometrial crypt covered by an intact epithelium. Both the non-hyphal stage (arrows) and hyphal stage (arrowheads) of *C. kefyr* are seen. Cow No. 3, PAS, bar = 50 µm

Microscopy of the cytology slides stained with Hemacolor^®^ (Merck Life Science A/S, Hellerup, Denmark) revealed the presence of numerous basophilic organisms with a diameter of around 1-3 µm located either as isolated organisms or as clusters. Many macrophages that had phagocytised numerous organisms were present (Fig. 1). The morphology of the organisms was consistent with yeast cells. The smear of cow No. 3 contained the most organisms. Neutrophils were only present in the cytology smear of cow No. 1 (84.1% neutrophils out of 200 cells counted). Histology of haematoxylin and eosin (HE) and periodic acid-Schiff (PAS) stained endometrial tissue sections of cow No. 2 revealed an intact endometrial epithelium covering a stroma diffusely infiltrated with mononuclear cells and with scattered neutrophils migrating towards the endometrial surface. Fungi were not observed. Histology of an endometrial biopsy of cow No. 3 revealed the presence of abundant numbers of mostly non-invasive PAS-positive organisms on the endometrial surface, especially in the endometrial crypts. Although most organisms were present as non-hyphal cells, fungal hyphae were occasionally seen of which some invaded the outmost part of the epithelium (Fig. 2). In the endometrium, areas of subepithelial fibrosis and a mild non-suppurative inflammation consisting of accumulation of cells around the superficial segments of the crypts as well as subepithelially was seen. Inflammation closely associated with the presence of fungi was not observed in any of the cows. The biopsy of cow No. 1 was of poor quality and not suitable for examination.

Due to the presence of numerous yeast-like organisms detected by microscopy, the lavage samples, which had been kept stored at −20 °C, were thawed and inoculated on (1) Sabouraud dextrose agar (Oxoid CM0041) and incubated for 2 weeks at 25 °C in atmospheric air and (2) Blood agar (Blood agar base (Oxoid CM0055) supplied with 5% sterile bovine blood) and incubated aerobically and anaerobically at 37 °C for 2 days. However, organisms were not cultured.

DNA was extracted from the three endometrial biopsies and from endometrial biopsies taken at random from ten cows (controls) from the same herd by using the DNeasy kit (Qiagen, Hilden, Germany) according to the protocol of total DNA extraction from plant tissue. From these samples, 597 base pair (bp) fragments of 18S rRNA were amplified using the universal fungus-specific primers NS3 (5′-GCAAGTCTGGTGCCAGCAGCC-3′) and NS4 (5′-CTTCCGTCAATTCCTTTAAG-3′) [10]. The universal plastid marker of eukaryotic algae and cyanobacteria were targeted with the primers p23SrV-F (5′-GGACAGAAAGACCCTATGAA-3′) and p23SrV-R (5′-TCAGCCTGTTATCCCTAGAG-3′) [11].

Polymerase chain reaction (PCR) analysis was carried out in a 50 μL reaction mix, which contained 1× Amplitaq Gold buffer (Thermo Fischer Scientific, Waltham, MA USA), 400 μM of each deoxynucleoside triphosphate (Amersham Biosciences, Piscataway, NJ), 0.4 μM of each primer, 2.5 U of AmpliTaq Gold^®^ DNA polymerase (Thermo Fischer Scientific), and 5 μL of template DNA. Thermal cycling using a T3 thermocycler (Biometra, Göttingen, Germany) was performed as follows: denaturation at 94 °C for 6 min, followed by 35 cycles of denaturation at 94 °C for 45 s, annealing at 55 °C for 45 s, and extension at 72 °C for 3 min, followed by a final elongation step of 10 min. The presence and purity of the product was determined by electrophoresis in a 1.5% agarose gel.

All the samples were negative with the universal algae primers. From the sample of cow No. 3, a clear PCR amplicon was generated with the fungi-specific primers. The positive PCR product was sequenced by cycle sequencing on an ABI 3130 genetic analyser (Applied Biosystems, Foster City, CA) using a BigDye Terminator v3.1 cycle sequencing kit (Applied Biosystems) according to the manufacturer’s instructions. Sequence analysis was performed with BioNumerics, version 7.1 (Applied-Math, Sint-Martens-Latem, Belgium). A single stranded sequence of 495 bp was generated with the fungi-specific primers. For identification of the closest relatives, the sequence of the unidentified insert was compared against sequences in GenBank (https://www.ncbi.nlm.nih.gov/) and proved to be 100% homologous to *K. marxianus*, i.e. the sexual (teleomorph) stage of *C. kefyr* [8]. However, the sequence also closely resembles other members of the *K. marxianus* group, as it was 99% homologous (corresponding to a difference in two bp) to *K. lactis* and *K. wickerhamii*.

Fluorescence in situ hybridization (FISH) examination of endometrial tissue sections of cow Nos. 2 and 3 was performed as previously described [12] to investigate the possible role of the *C. kefyr* and to confirm the identity of this organism. Probes targeting the overall bacterial domain Bacteria [13], fungi [14], chlorofyl algae [12] and the *K. marxianus* group were used (Table 1). The *K. marxianus* group is a monophyletic group of fungi and consists of the five closely related subspecies *K. marxianus, K. aestuarii*, *K. dobzhanskii*, *K. lactis*, and *K. wickerhamii* [15].

**Table 1:** Oligonucleotide probes targeting bacterial 16S rRNA and 18S rRNA of fungi and algae used for fluorescence in situ hybridization

Target	Name	Target sequence (5′–3′)	Labelling	Target region
Domain bacteria	S-D-Eub-338	5′ gct gcc tcc cgt agg agt 3´	FITC	16S
Fungi	D223_Svampe_Gen	5′ cca ccc act tag agc tgc 3′	Cy3	18S
Chlorofyl algae	CHLO	5′ gtg gtg gtc cgc acc tcg 3′	Cy3	18S
*Kluyveromyces marxianus* group^a^	Kluyv_620	5′ gaa aac cag tgc gcg aca t 3′	Cy3	18S

^a^Designed in PRIMROSE [16]

**Fig. 3:**
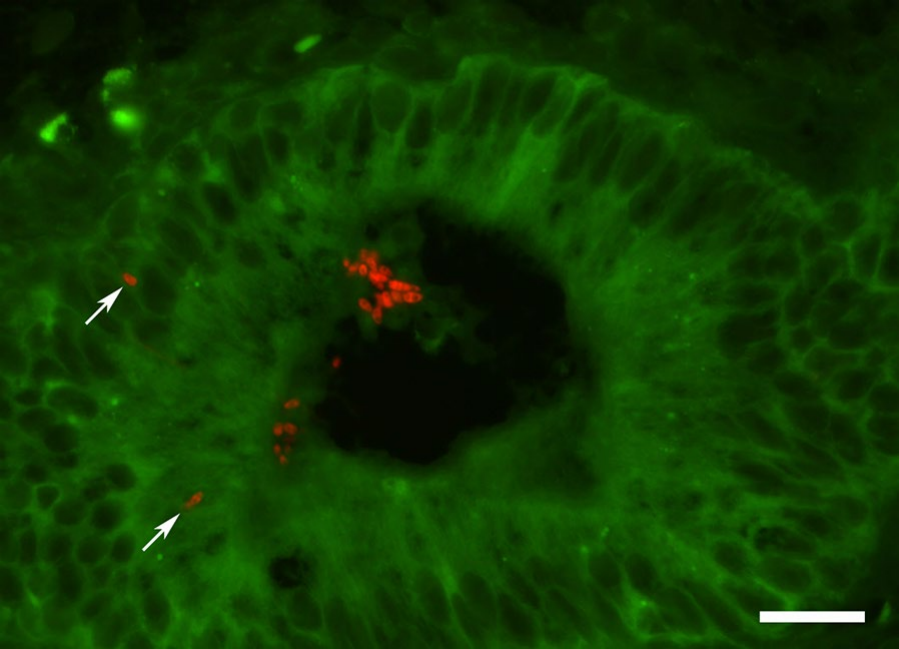
Flourescence photomicrograph showing *Candida kefyr* organisms in an endometrial biopsy. Multiple *C. kefyr* organisms (*red flourescence*) are located in the lumen of an endometrial crypt and a few organisms have invaded the epithelium (arrow). Cow No. 3, FISH, bar = 20 µm

FISH examination revealed the presence of *C. kefyr* in the two cows. The organisms occurred either as clusters or as isolated organisms located mainly on the endometrial surface, although isolated organisms were present within the superficial part of the epithelium (Fig. 3). The organisms were also detected by the probe targeting the fungal genus. Bacteria or algae were not detected by FISH.

Cow Nos. 1 and 2 were reexamined 21 days later by cytology of a uterine lavage sample and by histology and FISH of an endometrial biopsy, but infection with *C. kefyr* or other fungi was not found. Other laboratory findings are included in Additional file 1. Cow No. 1 was culled 137 days pp without having been inseminated (planned culling) and without further examination. Cow No. 2 became pregnant after three inseminations and calved with a normal calf.

Cow No. 3 was slaughtered 201 days after the detection of the *C. kefyr* infection and examined post mortem by cytology, histology and bacteriology without detection of the organism or the cause of infertility (data not shown).

## Conclusions

Microbial contamination of the uterine lumen and endometrial surface after calving develops due to migration through the cervical canal of microorganisms present on the mucosa of the post-cervical part of the genital tract. Different fungi, such as *Penicillium* sp. and *Candida albicans*, may be present in the cervico-vaginal mucosa of cows [17] and fungi may therefore participate in the colonization of the uterine lumen during the pp period.

Mycotic endometritis may develop under certain favorable conditions although this has not been reported in cattle. Attempts to induce uterine diseases by intrauterine inoculation of approximately 1 × 10^6^ *Aspergillus fumigatus* spores at breeding have failed [18]. However, repeated uterine manipulations and antibiotic therapy of non-pregnant mares has been reported to facilitate uterine infection with *A. fumigatus* and *C. albicans* [19]. In the present cases, a monoculture of *C. kefyr* was present in the uterus around 30 days pp in two cows and 7 months pp in one cow. It remains unknown how these infections became established as a monoculture, especially because the cows had not been treated with antibiotics and because a compromised immune system would probably have been reflected by the presence of a severe bacterial infection, i.e. metritis.

Although the fungi seemed not to invade the endometrium, but restricted their growth to the mucosal lining and luminal content, significant numbers of macrophages having phagocytised fungal elements were present in the luminal content. A significant neutrophilic inflammation was only present in Cow No. 1, but this was probably a part of the uterine inflammatory response often seen in pp cows and not induced by the fungi. Endometrial inflammation was present but due to its non-granulomatous character and not being closely associated with fungal elements, this was probably not induced by the fungi. As the fungi were mainly present in the non-hyphal stage, tissue invasion by hyphae, which is associated with necrotizing and granulomatous inflammation, was probably not of significance. The infections probably resolved spontaneously as no specific treatment were initiated as also observed in equine mycotic endometritis [19]. One of the cows became pregnant and delivered a normal calf at term, while the two others were not bred.

*Candida kefyr* is commonly isolated from milk of cows with mastitis [20], but has not been reported in association with other diseases of cattle although other fungi have been found in cases of systemic candidiasis [21]. However, *C. kefyr* has gained increasing attention in humans as a possible emerging pathogen associated with haematogenous infections [22]. It is remarkable that three cases were found in a single cattle herd by chance. This was only done because lavage samples were examined by microscopy as part of a research project. While examination of endometrial cytology and histology is widely used in equine theriogenology, these methods are usually not applied in cows under field conditions. The prevalence of *C. kefyr* colonization of the bovine uterus is therefore unknown, but the organism should be kept in mind when studying bovine uterine infections.

Examination of endometrial tissues by FISH proved very useful to confirm the sequencing findings, especially because the fungi could be visualized in situ. The organisms were localized on the endometrial surface and within the epithelium thus indicating a rather superficial infection. The colonization may allow self-recovery as reported in mares [19] but further research is needed to definitively conclude this. It is recommended to include histology when performing research on bovine uterine infections as tissue localisation may be important to determine the significance of microorganisms in a complex microbial environment such as the bovine uterus [9, 23].

## Additional file

**Additional file 1.** Laboratory data obtained from cytology of uterine flush samples and endometrial biopsies of cow Nos. 1 and 2 taken before and after the identification of the *Candida kefyr* infection (at postpartum (pp) days 27 and 29, respectively) as part of a larger research project on bovine pp endometritis.
