# Supplementary material for: Colonization of the bovine uterus by Candida kefyr
Source: Acta Vet Scand. 2017 Sep 16;59:61. doi: 10.1186/s13028-017-0329-5 (PMC5603010; doi:10.1186/s13028-017-0329-5)
Supplement: Supplementary file 1 — Additional file 1. Laboratory data obtained from cytology of uterine flush samples and endometrial biopsies of cow Nos. 1 and 2 taken before and after the identification of the Candida kefyr infection (at postpartum (pp) days 27 and 29, respectively) as part of a larger research project on bovine pp endometritis. [file 13028_2017_329_MOESM1_ESM.docx]

**Additional file 1** Laboratory data obtained from cytology of uterine flush samples and endometrial biopsies of cow Nos. 1 and 2 taken before and after the identification of the *Candida kefyr* infection (at postpartum (pp) days 27 and 29, respectively) as part of a larger research project on bovine pp endometritis.

| **Cow No.** | **Sampling**  **day pp** | **Uterine flush cytology** | | **Endometrial biopsy** | | |
| --- | --- | --- | --- | --- | --- | --- |
|  |  | **% neutrophils** | **Cytology findings** | | | **Bacteria detected by FISH*** |
| 1 | 6 | 89.0 | Epithelial cells, neutrophils, lymphocytes and macrophages | | *T. pyogenes* | |
|  | 48 | 0.0 | Epithelial cells | | None | |
| 2 | 8 | 58.5 | Epithelial cells, neutrophils, lymphocytes and macrophages | | None | |
|  | 50 | 0.0 | Epithelial cells, neutrophils and lymphocytes | | None | |

* FISH: Fluorescence *in situ* hybriddization using probes targeting *Fusobacterium necrophorum*, *Porphyromonas levii*, *Trueperella pyogenes* and *Escherichia coli.*
